# Design and synthesis of a fluorescent probe based on naphthalene anhydride and its detection of copper ions

**DOI:** 10.1371/journal.pone.0186994

**Published:** 2017-10-26

**Authors:** Guangjie He, Chenxi Liu, Xiaobo Liu, Qingzhi Wang, Aiying Fan, Songjun Wang, Xinlai Qian

**Affiliations:** 1 Department of Forensic Medicine, Xinxiang Medical University, Xinxiang, Henan Province, P. R. China; 2 Hebei Key Laboratory of Forensic Medicine, Hebei Medical University, Shijiazhuang, Hebei Province, P. R. China; 3 School of Basic Medical Science, Xinxiang Medical University, Xinxiang, Henan Province, P. R. China; Chinese Academy of Sciences, CHINA

## Abstract

Copper, as the third most abundant transition metal ions of human, plays an essential role in the redox reaction, signal transduction, hematopoiesis, and other physiological processes. Abnormal content of copper ions in the body will cause some diseases such as anemia, coronary heart disease, Menkes’ syndrome. In this article, a new fluorescence probe L for Cu^2+^ was designed and synthetized by using 4-bromo-1,8 naphthalene anhydride and 2-thiophene formaldehyde as raw materials. Fluorescent probe L itself exhibited strong fluorescence, upon the addition of Cu^2+^ ions, the fluorescence was quenched. The fluorescent detection limit for Cu^2+^ ions was determined to be 1.8 μM based on a 3δ/S method. UV-vis absorption and fluorescence spectra indicated that probe L showed good selectivity and sensitivity for Cu^2+^, and this selectivity was not interfered by other metal ions and anions. Further cell fluorescence imaging experiments indicated that the probe L had potential to be used to examine copper ions in vivo.

## 1. Introduction

Copper is the third most abundant transition metal ions after iron and zinc in the human body and plays essential roles in various physiological processes, such as participating in redox reactions, signal transduction and hematopoiesis, affecting the function of central nervous system, immune system and endocrine system. [1–3] Under normal circumstance, the copper ions in the biological system maintain normal physiological function, and keep a dynamic balance in the process of absorption, distribution, metabolism and excretion. The average normal concentration range of blood copper is 100~150 μg/L (15.7~23.6 μM). [4] When the exogenous copper is absorbed excessively or endogenous copper degradation is disturbed, homeostasis disequilibrium of copper ions occurs, causing many diseases such as anemia, coronary heart disease, Menkes’ syndrome, Wilson’s syndrome and Alzheimer's disease. [5–9] Therefore, it has attractive much interest for human health and environmental science to develop a sensitive method to selectively detect and quantify Cu^2+^ levels in aqueous solution and in vivo.

Among the variety of methods for detecting copper ions, [10–14] fluorescence analysis methods have received more attention due to their high fluorescence quantum yield, long analytical wavelength, good biocompatibility and stability, high selectivity and sensitivity and wide dynamic response range. They could also realize the sensitive and in situ real-time imaging of living body, which has achieved widely use in the field of cell biology and molecular biology. [15–18] Till now, many examples of fluorescence probes for Cu^2+^ ions have been reported [19–22], but there are many problems need to be improved, such as complexity synthesis steps, narrow pH range, low water solubility, poor sensitivity and selectivity. To overcome these problems, the development of simple molecular probes for Cu^2+^ metal ions that are cost-effective, rapid and high sensitive, is still in great demand.

1,8-naphthalimide, as a kind of large conjugated fluorescent molecules with a rigid plane, is easily modified. These compounds exhibit excellent properties in terms of photochemical stability, fluorescence quantum yield and stokes shift, which are widely used in laser dyes, fluorescent probes, DNA intercalators, solar cells and cancer treatment.[23–27] Moreover, Schiff base-type compounds are efficient metal chelators, which are widely employed for the construction of fluorescence probe.[28–30] In this paper, a new fluorescent probe L with Schiff base structure was synthesized by using naphthalimide derivative as fluorescent group. Fluorescence probe L exhibited strong fluorescence, and after coordination with Cu^2+^ ions, the fluorescence of L was quenched. The probe L showed a high sensitivity and selectivity for Cu^2+^, and has potential application in real-time imaging in living cells.

## 2. Experiment section

### 2.1 Materials and reagents

4-bromo-1,8-naphthalic anhydride and 2-thiophene formaldehyde were purchased from Tianjin Heowns Biochemical Technology Co., Ltd., 85% hydrazine hydrate was purchased from Sinopharm Shanghai Chemical Reagent Company, N-butylamine was purchased from Tianjin Kermel Chemical Reagent Co., Ltd., Chromatography of pure dimethyl sulfoxide (DMSO), 4-hydroxyethylpiperazineethanesulfonic acid (HEPES buffer), absolute ethanol, acetonitrile, dichloromethane, N,N-dimethylformamide (DMF) are all commercially available analytical reagents. Perchlorate solutions (2.0×10^−2^ M) of various metal ions (Al^3+^, K^+^, Na^+^, Mg^2+^, Ca^2+^, Cr^3+^, Mn^2+^, Fe^2+^, Fe^3+^, Co^2+^, Ni^2+^, Zn^2+^, Cd^2+^, Hg^2+^, Ag^+^, Pb^2+^, Cu^2+^) and stock solutions (2.0×10^−2^ M) of the anions (F^-^, Cl^-^, Br^-^, I^-^, NO_3_^-^, SO_4_^2-^, SO_3_^2-^, HSO_3_^-^, PO_4_^3-^, HPO_4_^2-^, H_2_PO_4_^-^, CO_3_^2-^, HCO_3_^-^, CH_3_COO^-^, PPi, SCN^-^, and S^2-^) were prepared in aqueous solutions, and diluted before use.

### 2.2 Instruments

^1^H NMR and ^13^C NMR spectra were performed on a Bruker Ascend™ 400 spectrometer with chemical shifts reported as ppm with TMS as internal standard. Mass spectrometric data were carried on with a Bruker Microtof-QIII spectrometry. UV-vis absorption spectra were measured with Shimadzu UV2600 spectrophotometer. Fluorescence spectra were recorded with Edinburgh Instruments FS-5 fluorescence spectrophotometer. IR spectral data were recorded with PerkinElmer FT-IR Spectrometer. Cell imaging was recorded with Leica DMI8 inverted fluorescence microscope.

### 2.3 Methods

Stock solutions of compound L (10 mM) for spectral measurement were prepared in CH_3_CN:HEPES (3:2, v/v) solution. Stock solutions of compound L for fluorescence imaging in cells were prepared in DMSO solution. Each time a 3 mL compound L was filled in a quartz cell of 1 cm optical path length, and different stock solutions of metal ions were added into the quartz cell gradually by using a micro-syringe.

### 2.4 Synthetic procedures

**Fig 1:**
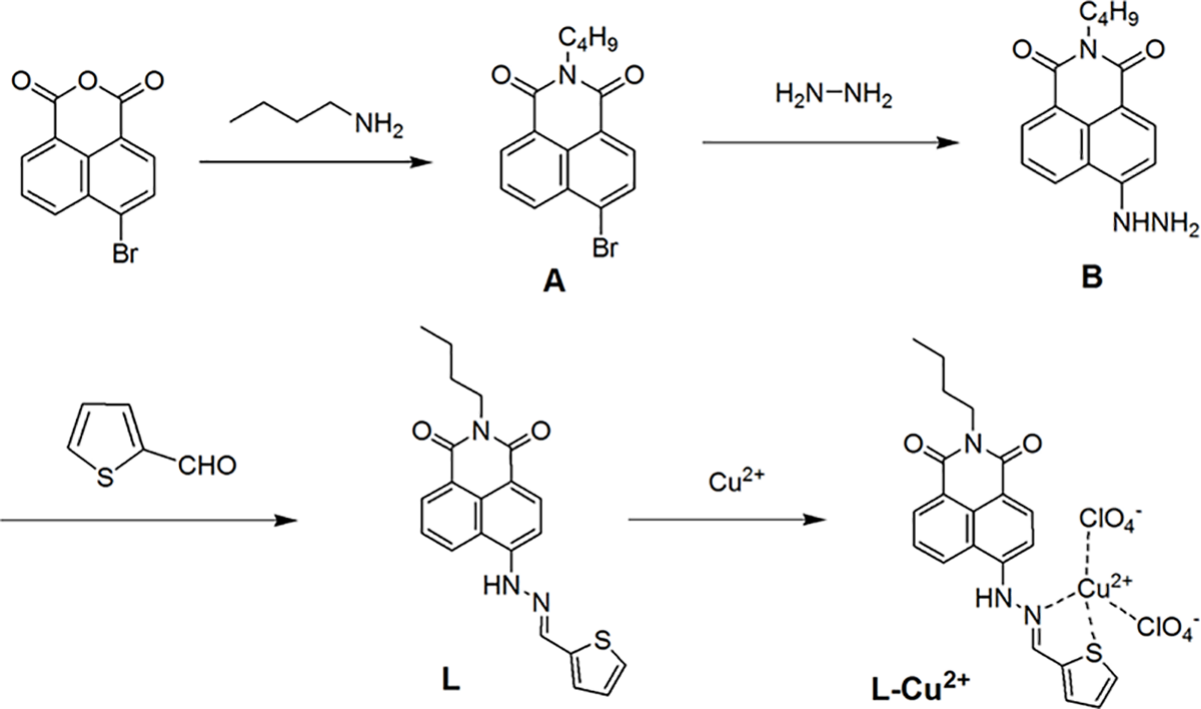
Syntheses of the fluorescent probe L and proposed binding mode of L to Cu^2+^.

The synthetic procedures for fluorescent probe L and proposed binding mode of L to Cu^2+^ ions was shown in Fig 1.

#### 2.4.1 Preparation of (6-Bromo-2-butyl-benzo[de]isoquinoline-1,3-dione) (A) [31]

4-bromo-1,8 naphthalic anhydride (0.7010 g, 2.53 mmol) was dissolved in 35 mL of ethanol. A solution of N-butylamine (0.1997 g, 2.73 mmol) in 5 mL of ethanol was added dropwise with stirring. The mixture was refluxed for 8 h and cooled to room temperature to precipitate a large amount of solids. The crude product was recrystallized from ethanol to give pure product A, yield 91.3%. M. p.: 103–104 °C.

#### 2.4.2 Preparation of 6-hydrazino-benzo[de]isoquinoline-1,3-diones (B)

A solution of 6-Bromo-2-butyl-benzo[de]isoquinoline-1,3-dione (6.0459 g, 18.2 mmol) and 20 mL methoxyethanol containing 2 mL 80% hydrazine hydrate was added to a 50 mL single-necked flask. The mixed solution was refluxed for 3 h and cooled to room temperature to precipitate a large amount of deposition. Filtered and washed several times with ethanol to give a yellow crystalline product B, yield 85%. M. p.: 220–222 °C.

#### 2.4.3 Preparation of the fluorescent probe L

6-hydrazino-benzo[de]isoquinoline-1,3-diones B (0.1416 g, 0.5 mmol) was dissolved in 20 ml of methanol and 2-thiophene formaldehyde (0.1122 g, 1.0 mmol) was added. Under nitrogen, the mixture was heated and refluxed for 12 hours. After cooling and filtration, the pure product L was obtained. The structural characterization of the fluorescent probe L was shown in the supplementary materials (S1–S3 Figs). ^1^H NMR (400 MHz, d_6_-DMSO) δ 11.47 (s, 1H), 8.77 (d, J = 8.4 Hz, 1H), 8.65 (s, 1H), 8.49 (d, J = 7.2 Hz, 1H), 8.39 (d, J = 8.5 Hz, 1H), 7.87–7.77 (m, 1H), 7.67 (d, J = 5.0 Hz, 1H), 7.59 (d, J = 8.5 Hz, 1H), 7.49 (d, J = 3.0 Hz, 1H), 7.17–7.16 (m, 1H), 4.09–3.98 (m, 2H), 2.51–2.50 (m, 2H), 1.39–1.30 (m, 2H), 0.93 (t, J = 7.3 Hz, 3H). ^13^C NMR (100 MHz, d_6_-DMSO) δ = 164.1, 163.4, 146.6, 139.9, 139.7, 134.0, 131.3, 130.3, 128.7, 128.5, 125.5, 119.1, 111.4, 107.1, 30.3, 20.3, 14.2. ESI-MS: m/z: 400.1078, [L+Na]^+^.

## 3. Results and discussion

### 3.1 The UV-vis spectra responses of probe L

**Fig 2:**
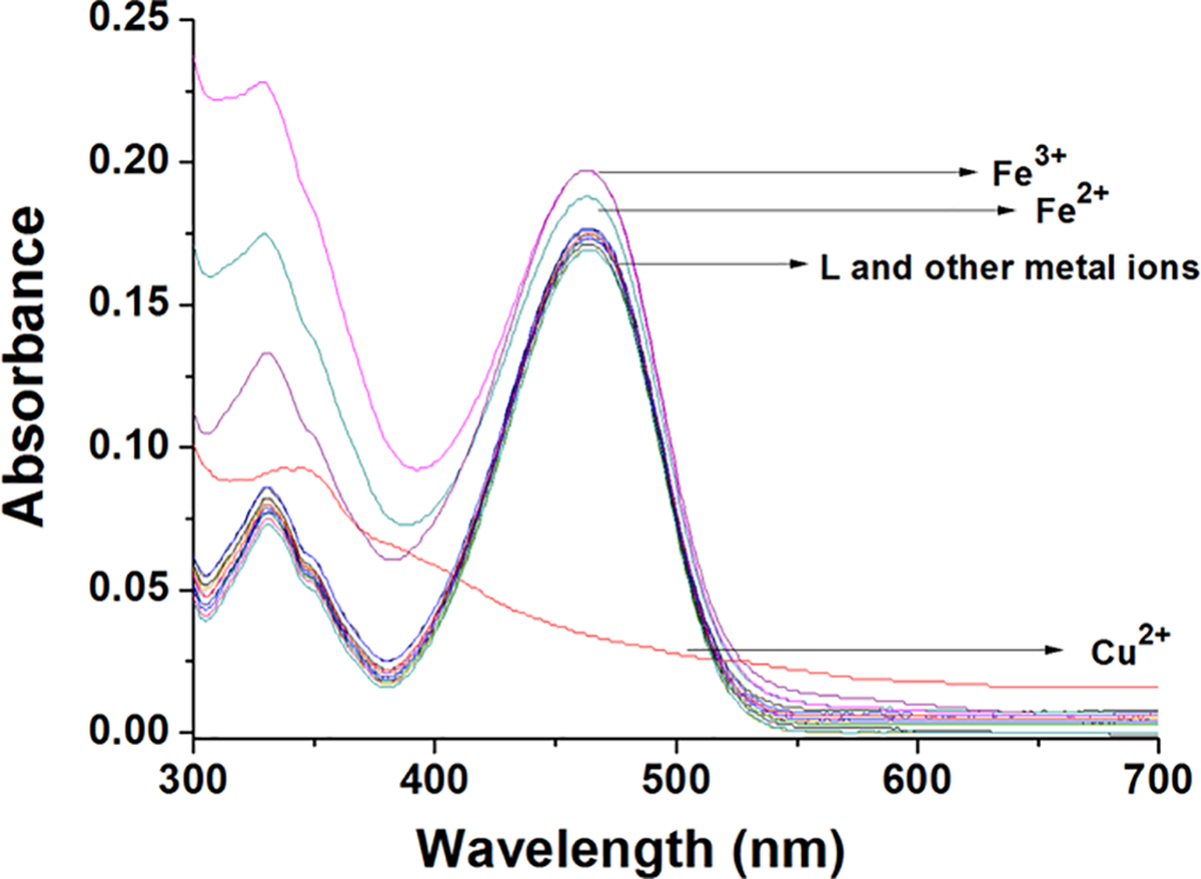
UV-vis spectra of the fluorescent probe L (10 μM) upon addition of various metal ions (4 equiv.) in CH_3_CN:HEPES (3:2, v/v, pH = 7.4).

A variety of metal ions had been added to the probe L (10 μM) in CH_3_CN: HEPES (3:2, v/v, pH = 7.4) solvents and their UV-vis spectra scanning was carried out. As shown in Fig 2, the UV-vis absorption peak around 465 nm was decreased when Cu^2+^ ions were added to the probe L solution. While adding other metal ions such as Ag^+^, Ca^2+^, Co^2+^, Cr^3+^, Fe^3+^, Fe^2+^, K^+^, Mg^2+^, Na^+^, Ni^2+^, Zn^2+^, Cd^2+^, Mn^2+^, Hg^2+^ and Pb^2+^, the UV-vis absorption of the fluorescent probe L didn’t have any significant changes. The results indicated that the probe L had a good selectivity for Cu^2+^ ions.

### 3.2 The UV-vis spectra titration of fluorescent probe L to Cu^2+^ ions

**Fig 3:**
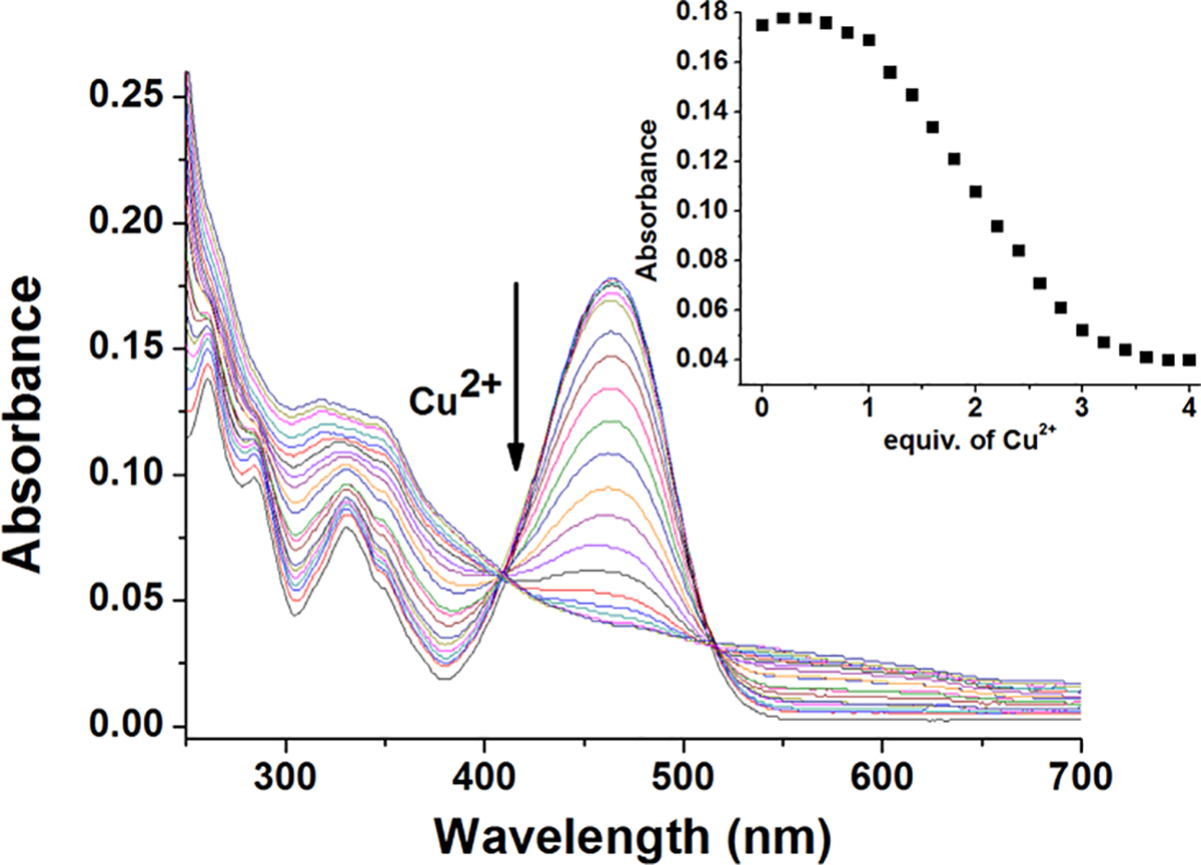
UV-vis spectra of fluorescent probe L (10 μM) upon addition of Cu^2+^ ions in CH_3_CN:HEPES (3:2, v/v, pH = 7.4) solution. Insert: UV-vis titration profile of the fluorescent probe L at 465 nm.

In the CH_3_CN:HEPES (3:2, v/v, pH = 7.4) solution, the UV-vis absorption spectra of the fluorescent probe L (10 μM) upon the addition of Cu^2+^ ions were shown in Fig 3. The maximum UV-vis absorption peak of fluorescent probe L itself was centred at 465 nm, which showed a broad naphthalimide-based π-π transition band. However, with the addition of Cu^2+^ ions, the UV-vis absorption peak at 465 nm of fluorescence probe L gradually decreased. When 4 equivalents of Cu^2+^ ions were added, the peak reached equilibrium. It was caused by a strong binding interaction between thiophene group of probe L and Cu^2+^. The stoichiometry of compound L and copper ions was determined to be 1:1 from the Job’s plot (S4 Fig).

### 3.3 The pH dependence of the probe

The pH value may influence the fluorescence properties of compound L and complex L-Cu^2+^, hence the pH-dependent measurements in CH_3_CN: HEPES (3:2, v/v, pH = 7.4) solution were performed. As shown in S5 Fig, the fluorescent intensity of both compounds showed no obviously change in the pH 4.0–10.0, which suggested that both the chemical structure and fluorescence properties of this probe were relatively stable over a broad range of pH values. Thus the pH range of the solution in the following tests was controlled at 7.4 and which also indicated that the probe could be applied in physiological condition.

### 3.4 The fluorescence titration of probe L to Cu^2+^ ions

**Fig 4:**
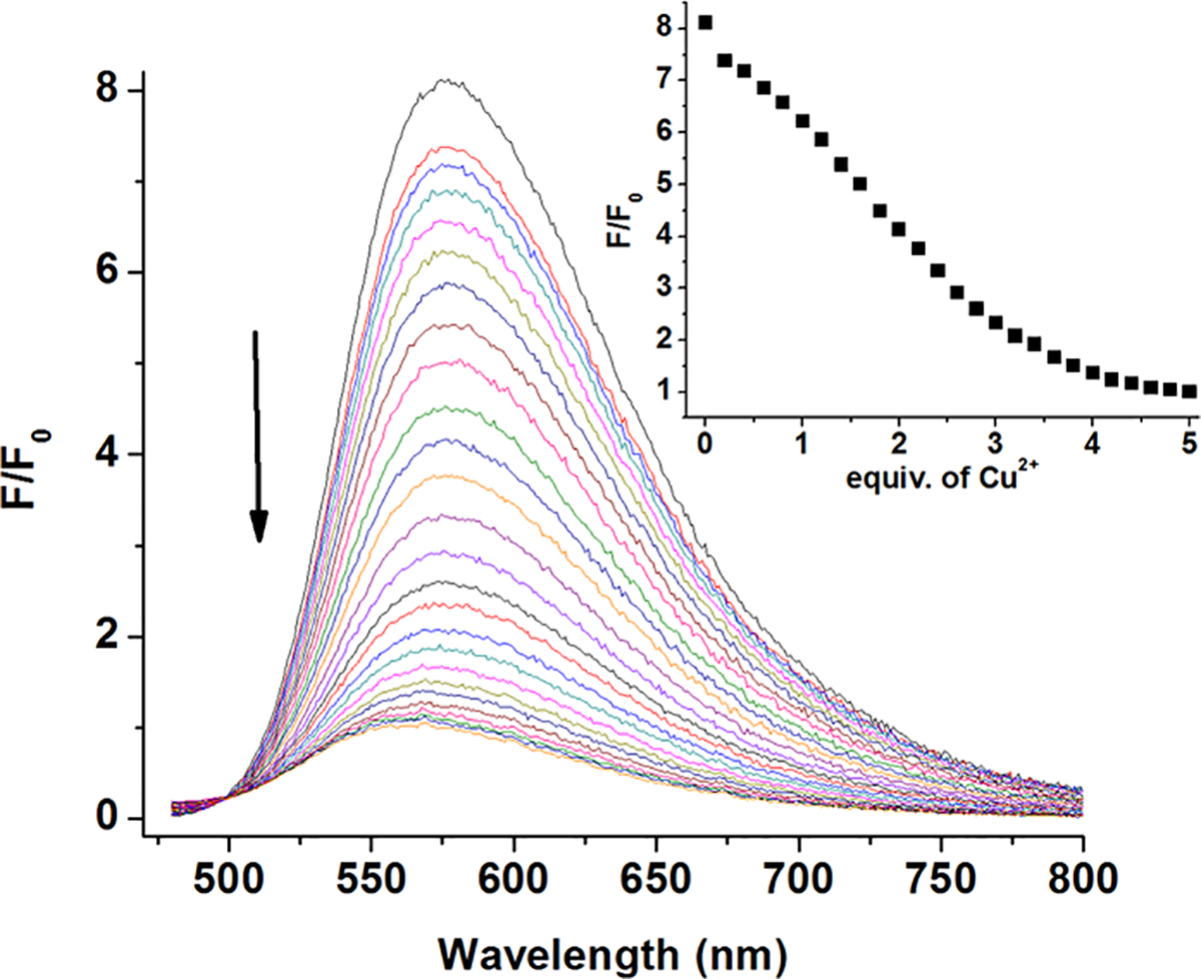
The fluorescence spectra titration of fluorescent probe L (10 μM) upon the addition of Cu^2+^ ions in CH_3_CN: HEPES (3:2, v/v, pH = 7.4) solution. Insert: Fluorescence titration profile of the fluorescent probe L at 575 nm (excited at 465 nm).

**Fig 5:**
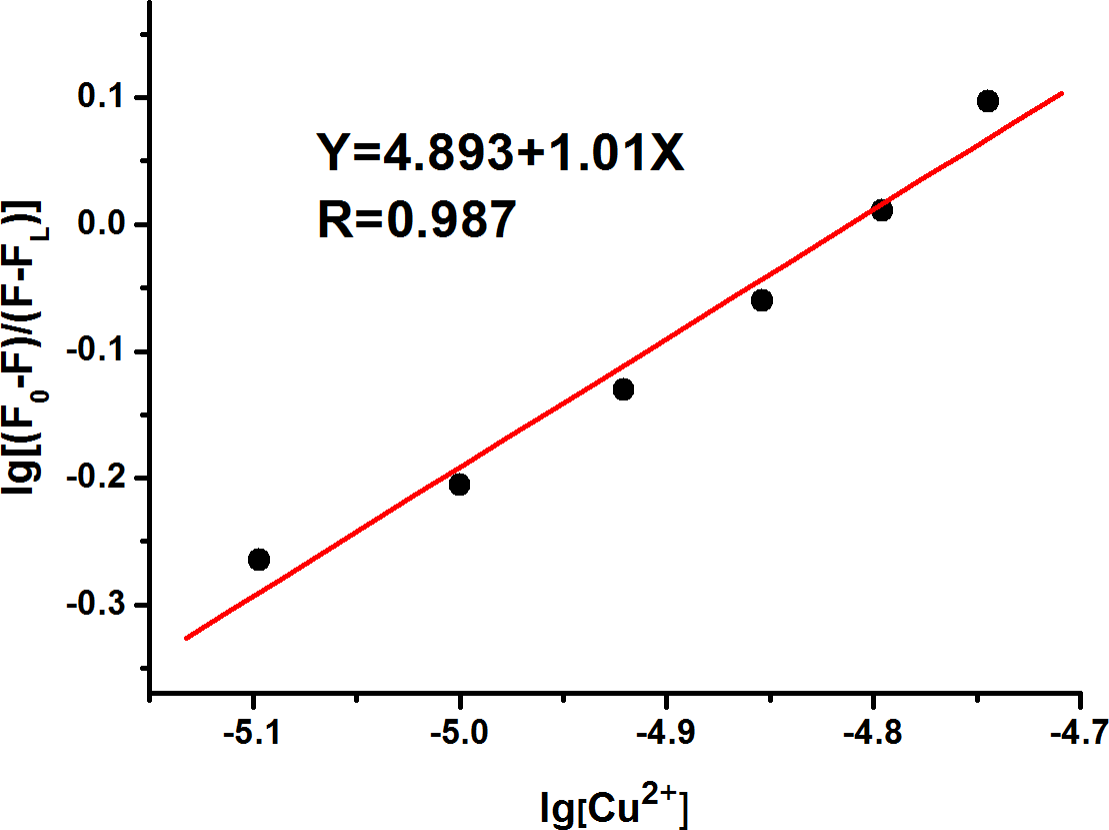
Benesi-Hildebrand linear fitting demonstrated a 1:1 stoichiometry for the L-Cu^2+^ complexation species.

In CH_3_CN: HEPES (3:2, v/v, pH = 7.4) solution, the fluorescence titration spectra of the fluorescent probe L (10 μM) to Cu^2+^ ions was shown in Fig 4. Excited at 465 nm, the fluorescence probe L showed a strong fluorescence emission at 575 nm. Upon the addition of Cu^2+^ ions, the fluorescence intensity of the probe L gradually decreased. When approximate 4 equivalents of Cu^2+^ ions were added, the fluorescence intensity decreased to a balance. The reduction of fluorescence intensity may be attributed to the d-d electron paramagnetic property and/or photo induced electron transfer of copper ions. [32,33] The Benesi-Hildebrand data analysis indicated that the fluorescence probe L and Cu^2+^ were combined with the 1: 1 coordination ratio (Fig 5), with an association constant (K) being calculated as K = 7.8×10^5^ M^-1^. To further investigate the sensitivity of the probe L for Cu^2+^, the detection limit was calculated based on the fluorescence titration. Subsequent data analysis displayed an excellent linear relationship (R = 0.993) between the fluorescence intensity at 575 nm and Cu^2+^ concentration from 4 to 12 μM (S6 Fig). The detection limit of Cu^2+^ is determined to be 1.8 μM based on the equation of LOD = 3δ/S, where δ is the standard deviation of blank measurements and S is the slope between fluorescence intensity versus Cu^2+^ concentration. It was lower than the limit of copper in drinking water set up by the U.S. Environmental Protection Agency (EPA). These results indicated that the fluorescent probe L had good sensitivity to Cu^2+^ ions. Moreover, the reversibility experiment of probe L was performed by addition of strong chelate group EDTA to the complex L-Cu^2+^ (S7 Fig). Upon addition of 100 equivalents of EDTA, there was no significant recovery for the fluorescent intensity, which indicated the irreversible feature of the probe L. From a mechanistic viewpoint, the high sensitivity of the probe for Cu^2+^ ions was likely attributed to many combined influences, including the suitable coordination conformation of the Schiff-based unit, the thiophene-affinity feature of the Cu^2+^ ions and the deprotonation ability of the Cu^2+^ ions.

### 3.5 Character of the complexation of probe L with Cu^2+^

The complexation formation of L with Cu^2+^ in the 1: 1 ratio was confirmed by ESI-MS spectra. As shown in S8 Fig, upon the addition of 1 equiv. Cu^2+^ metal ions to the solution of L in ethanol, the peak at m/z = 585.5319 corresponding to [L+Cu^2+^+ClO_4_^-^+CH_3_CH_2_OH+H]^+^ was observed. The specific isotopic patterns fit well with the simulated peaks calculated by IsoPro 3.0 program. IR spectrum of compound L-Cu^2+^ also revealed the possible coordination model of compound L with copper ions (S9 and S10 Figs). Compound L exhibited characteristic peak of -C = N- group at 1554.99 cm^-1^. After coordinated with copper ions, the characteristic stretching band of the -C = N- group in complex L-Cu^2+^ was shifted to 1590.50 cm^-1^, which was due to the participation in the coordination interaction.

### 3.6 The selectivity and competition experiments of probe L

**Fig 6:**
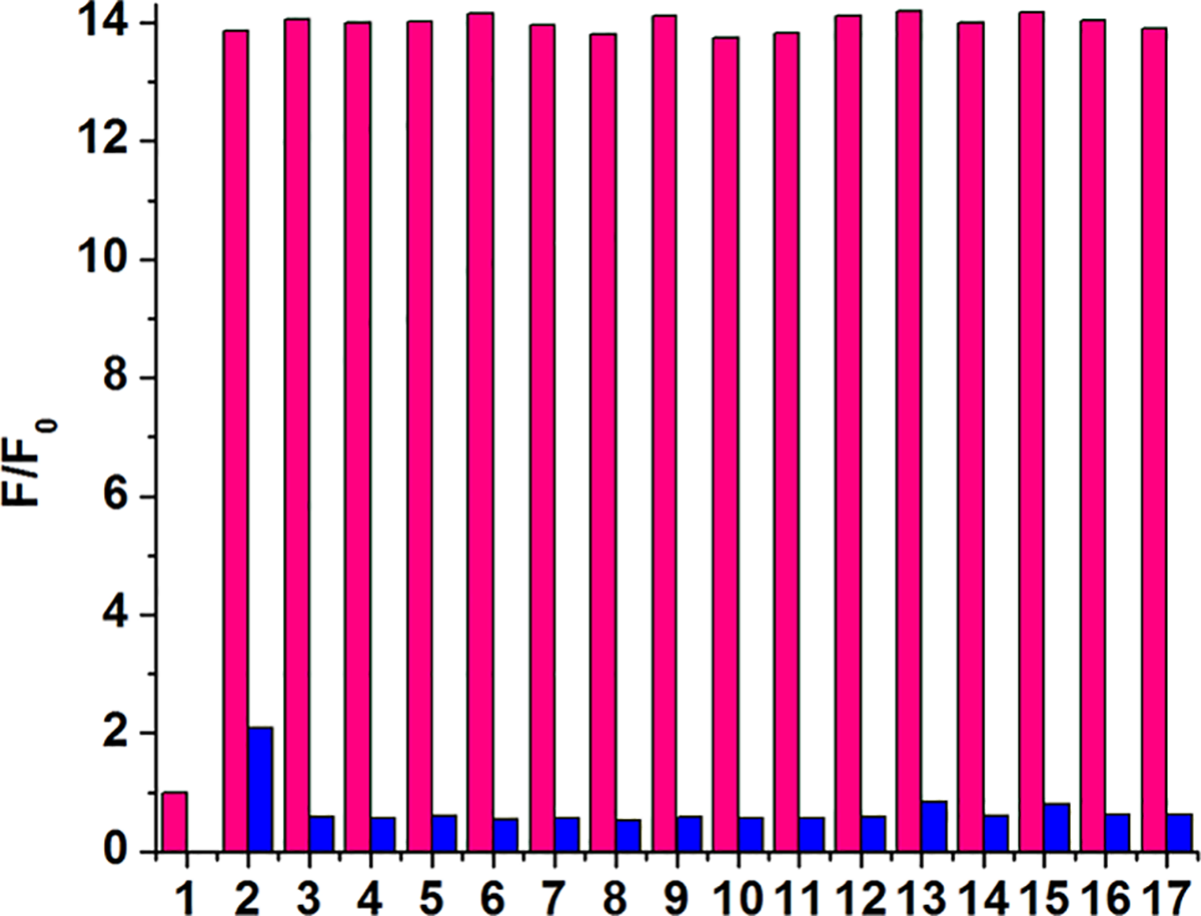
The fluorescence spectra selectivity of the fluorescent probe L (10 μM) upon the addition of various metal ions (4 equiv.) in CH_3_CN:HEPES (3:2, v/v, pH = 7.4) solution (pink bars): (1) Cu^2+^, (2) Mn^2+^, (3) Fe^2+^, (4) Fe^3+^, (5) Ni^2+^, (6) K^+^, (7) Cd^2+^, (8) Mg^2+^, (9) Na^+^, (10) Al^3+^, (11) Co^2+^, (12) Pb^2+^, (13) Hg^2+^, (14) Cr^3+^, (15) Ag^+^, (16) Ca^2+^, (17) Zn^2+^. The blue bars indicated the fluorescence intensity of L after adding Cu^2+^ ions to the above solutions except 1.

In order to further explore the selectivity of the probe L to Cu^2+^ ions, the fluorescence responses of L to different metal ions and anions were tested. As shown in Fig 6 and S11 Fig, in the CH_3_CN:HEPES (3:2, v/v, pH = 7.4) solution, various metal ions, such as Cu^2+^, Mn^2+^, Fe^2+^, Fe^3+^, Ni^2+^, K^+^, Cd^2+^, Mg^2+^, Na^+^, Al^3+^, Co^2+^, Pb^2+^, Hg^2+^, Cr^3+^, Ag^+^, Ca^2+^, Zn^2+^, were added respectively to the solutions of the fluorescence probe L (10 μM). Only after the addition of Cu^2+^ ions, the fluorescence intensity of the probe L was obviously reduced. When the other metal ions were added, the fluorescence intensity of the probe L remained nearly unchanged. However, with the subsequent addition of Cu^2+^ ions into the above solutions, the fluorescence intensity of L decreased obviously. In addition, the fluorescence response of probe L toward Cu^2+^ was also investigated in the presence of other competing anions such as F^-^, Cl^-^, Br^-^, I^-^, NO_3_^-^, SO_4_^2-^, SO_3_^2-^, HSO_3_^-^, PO_4_^3-^, HPO_4_^2-^, H_2_PO_4_^-^, CO_3_^2-^, HCO_3_^-^, CH_3_COO^-^, PPi, SCN^-^ and S^2-^. As shown in S12 Fig, after adding these anions to the solution of L-Cu^2+^, the fluorescent intensity of L-Cu^2+^ didn’t have any obvious change. All these results indicate that L has good selectivity to Cu^2+^ ions in CH_3_CN: HEPES (3:2, v/v) solution over other analytes mentioned above, which enable it to have potential applications for the detection of Cu^2+^ ions in living cells.

### 3.7 Cell fluorescence imaging of probe L for Cu^2+^ ions

**Fig 7:**
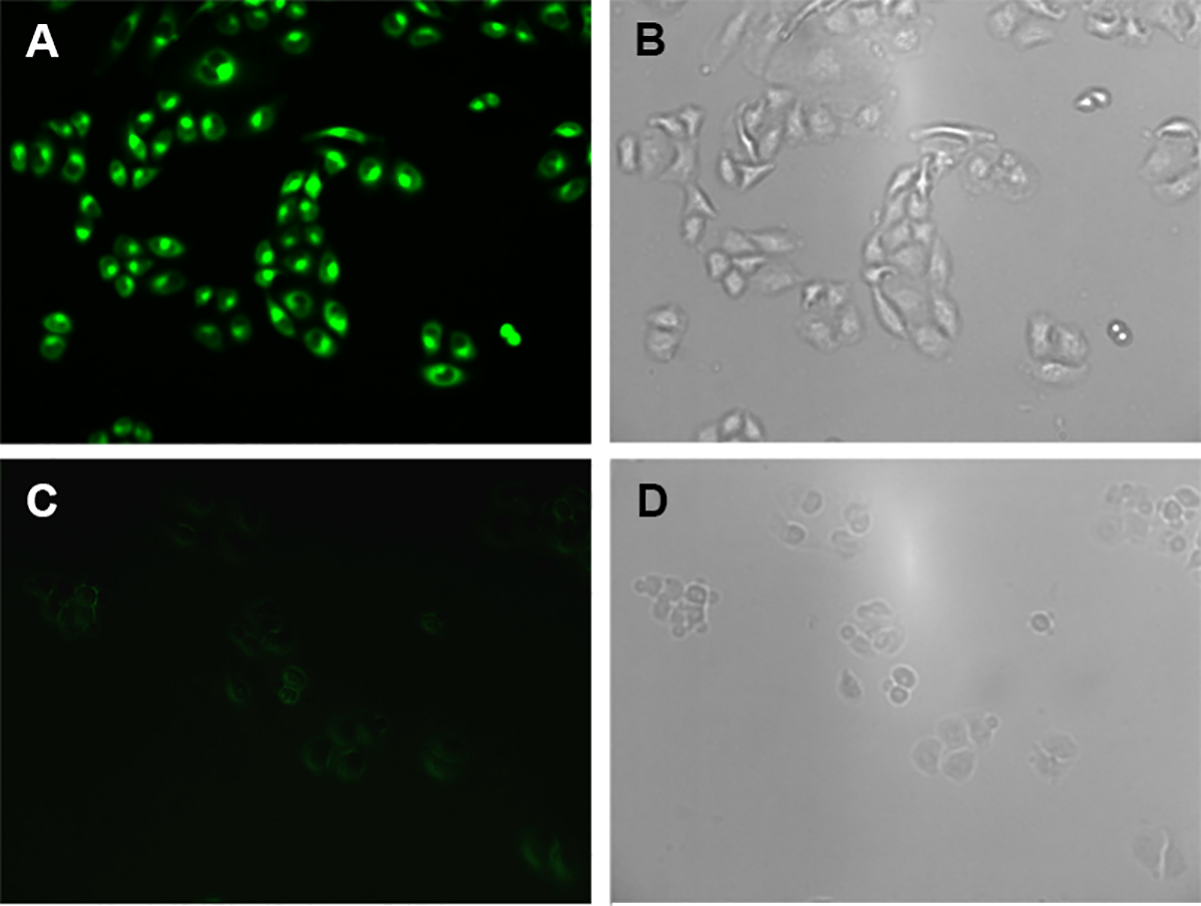
The fluorescence images of LO-2 cells incubated with probe L (3 μM) (A); probe L after addition of 5 equivalents of Cu^2+^ ions (C); and their corresponding bright field images (B and D).

The application of fluorescent probes in cell imaging is of great significance for biological applications. The fluorescence imaging of probe L was performed in normal hepatic cells LO-2. Prior to fluorescence imaging, LO-2 cells were cultured in a 12-well cell plate for 24 hours, and incubated with the probe L (3 μM) at 37 °C, 5% CO_2_ for 3 hours and then washed three times with PBS solution. Under Leica DMI8 inverted fluorescence microscope, the cell imaging results of LO-2 were shown in Fig 7. Excited with blue light, probe L showed significant intracellular green fluorescence in LO-2 cells, indicating that probe L had strong cell permeability. The green fluorescence in LO-2 cells was significantly quenched by adding 5 equivalents of copper ions. Fluorescence imaging experiments showed that the probe L had the capacity for the detection of copper ions in vivo.

## 4. Conclusion

In summary, we developed a new fluorescence probe based on naphthalimide and thiophene moiety for the detection of Cu^2+^ ions. The Job-plot fitting analysis indicated a 1:1 stoichiometry of probe L with Cu^2+^ ions. The results of UV-vis absorption and fluorescence spectra experiments showed that the fluorescent probe L possessed good selectivity to Cu^2+^ ions in CH_3_CN:HEPES (3:2, v/v, pH = 7.4) solution and could be used to detect Cu^2+^ ions without interference from other metal ions. Furthermore, cell imaging results indicated that L can visually trace intracellular Cu^2+^ ions in living cells, which would be helpful to design new probes in environmental and biological systems.

## Supporting information

S1 Fig ^1^H NMR spectrum of the fluorescent probe L (DMSO-d_6_). (TIFF)

S2 Fig ^13^C NMR spectrum of the fluorescent probe L (DMSO-d_6_). (TIFF)

S3 Fig ESI-MS spectrum of the fluorescent probe L. (TIF)

S4 Fig Job’s Plot analysis of L with Cu^2+^ in CH_3_CN:HEPES (3:2, v/v, pH = 7.4) solution (total concentration: 50 μM). (TIF)

S5 Fig The pH-dependent measurements of L (10 μM) in CH_3_CN:HEPES (3:2, v/v, pH = 7.4) solution with and without Cu^2+^ (4 equiv.). Fluorescence intensity was recorded at 575 nm.
(TIF)

S6 Fig Linearity of fluorescent response of L to Cu^2+^ in CH_3_CN: HEPES (3:2, v/v) solution with an excitation wavelength at 465 nm. (TIF)

S7 Fig The fluorescence spectra response of L-Cu^2+^ upon increasing addition (10, 20, 30, …100 equiv.) of EDTA-2Na in CH_3_CN:HEPES (3:2, v/v, pH = 7.4) solution. (TIF)

S8 Fig ESI-MS spectrum of the compound L-Cu^2+^. (TIF)

S9 Fig IR spectrum of the fluorescent probe L. (TIF)

S10 Fig IR spectrum of the compound L-Cu^2+^. (TIF)

S11 Fig The fluorescence spectra selectivity of the fluorescent probe L (10 μM) to various metal ions in CH_3_CN:HEPES (3:2, v/v, pH = 7.4) solution. (TIF)

S12 Fig The fluorescence spectra response of L-Cu^2+^ to various anion in CH_3_CN:HEPES (3:2, v/v, pH = 7.4) solution. (1)L, (2)L-Cu^2+^, (3)F^-^, (4)Cl^-^, (5)Br^-^, (6)I^-^, (7)NO_3_^-^, (8)SO_4_^2-^, (9)SO_3_^2-^, (10)HSO_3_^-^, (11)PO_4_^3-^, (12)HPO_4_^2-^, (13)H_2_PO_4_^-^, (14)CO_3_^2-^, (15)HCO_3_^-^, (16)CH_3_COO^-^, (17)PPi, (18)SCN^-^, (19)S^2-.^
(TIF)
