# Supplementary figures and images for: Design and synthesis of a fluorescent probe based on naphthalene anhydride and its detection of copper ions
Source: PLoS One. 2017 Oct 26;12(10):e0186994. doi: 10.1371/journal.pone.0186994 (PMC5658116; doi:10.1371/journal.pone.0186994)

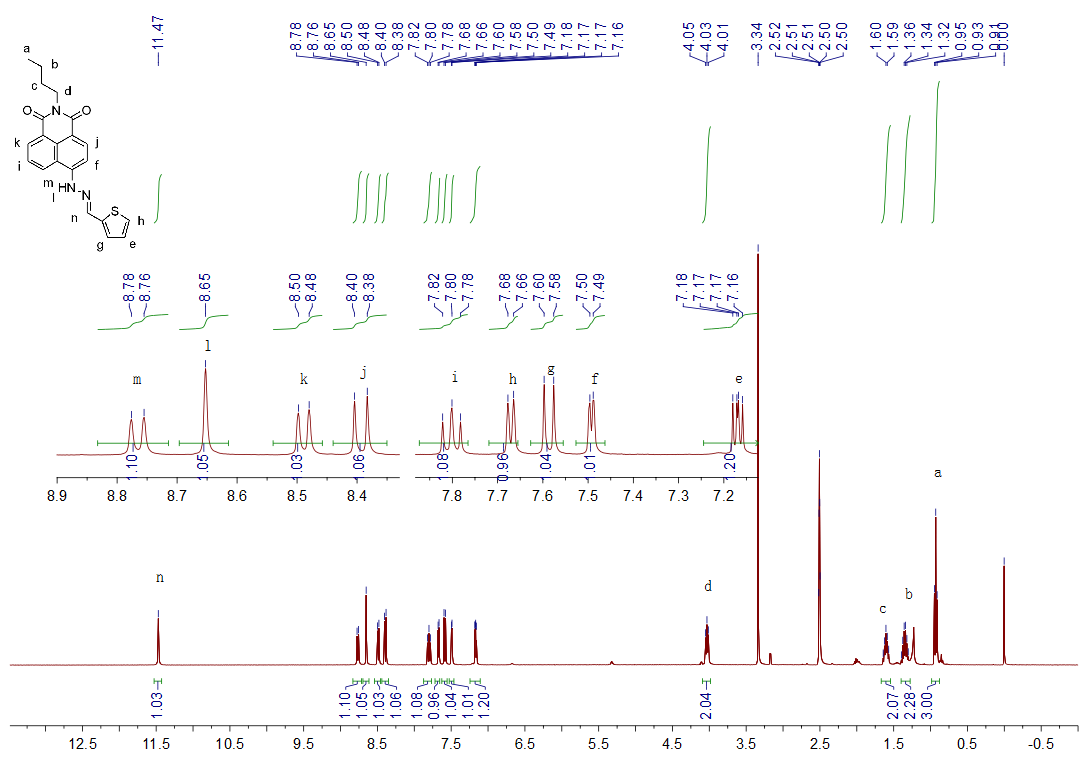

Supplement: S1 Fig — (TIFF) [file pone.0186994.s001.tiff]

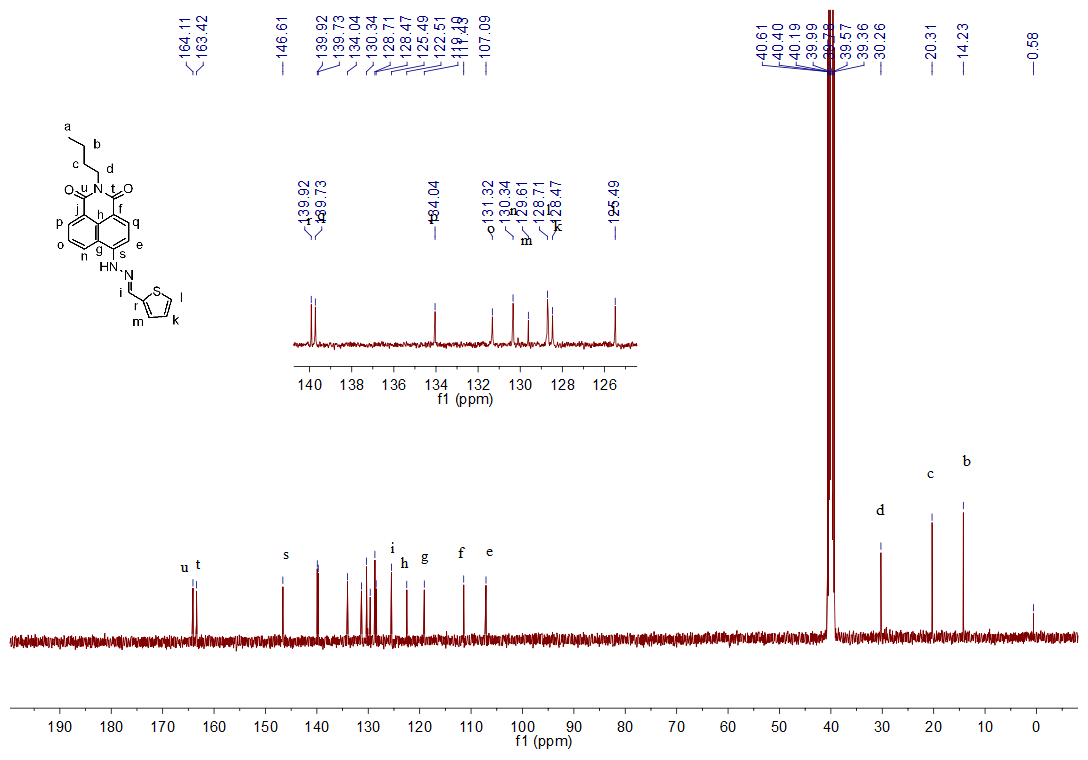

Supplement: S2 Fig — (TIFF) [file pone.0186994.s002.tiff]

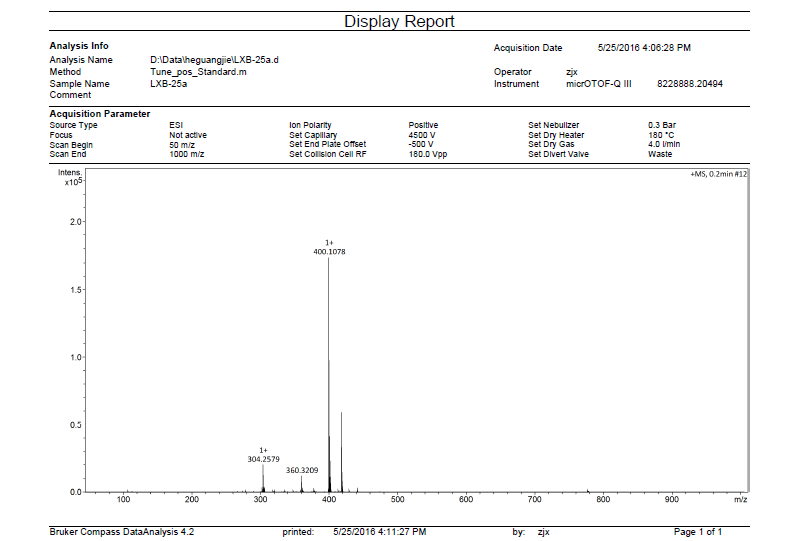

Supplement: S3 Fig — (TIF) [file pone.0186994.s003.tif]

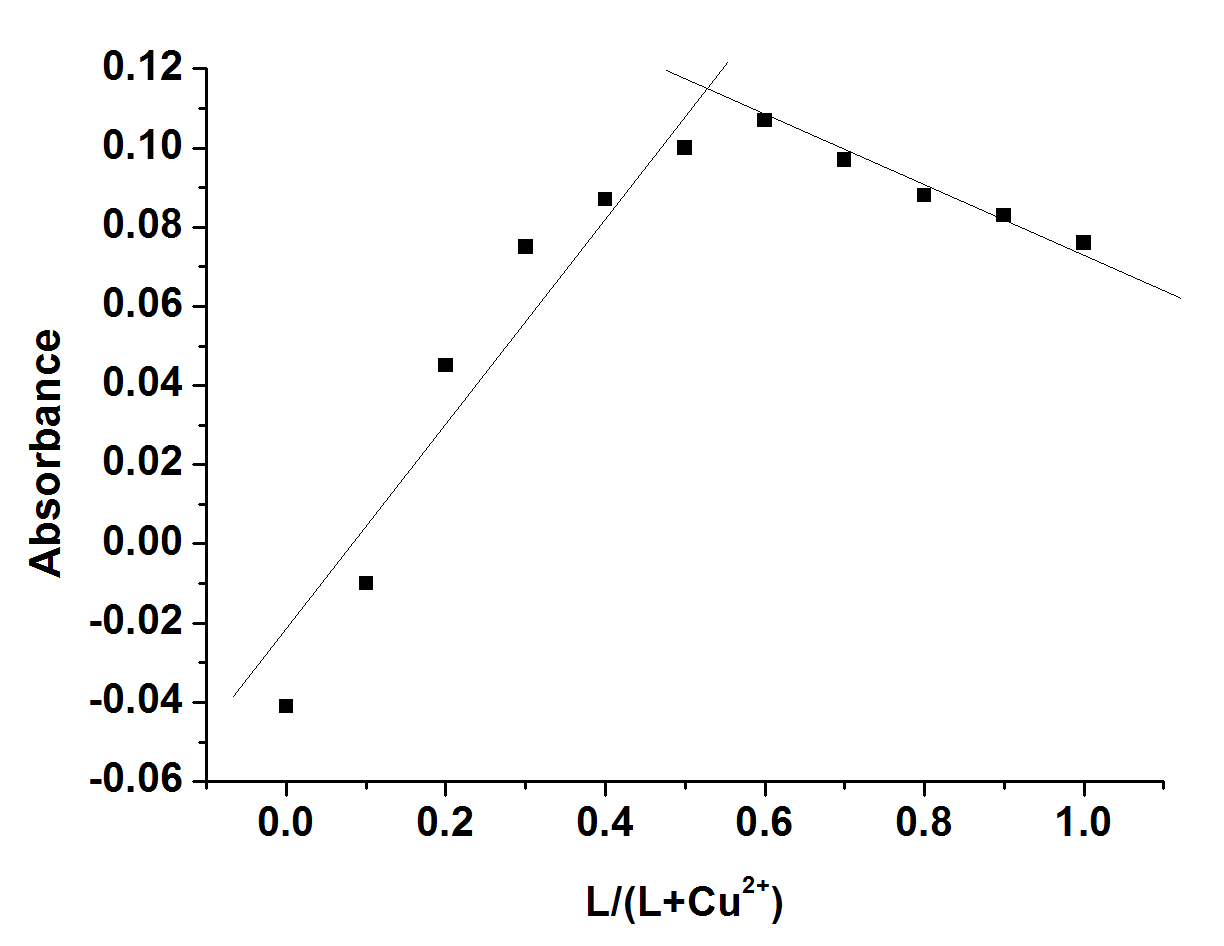

Supplement: S4 Fig — (TIF) [file pone.0186994.s004.tif]

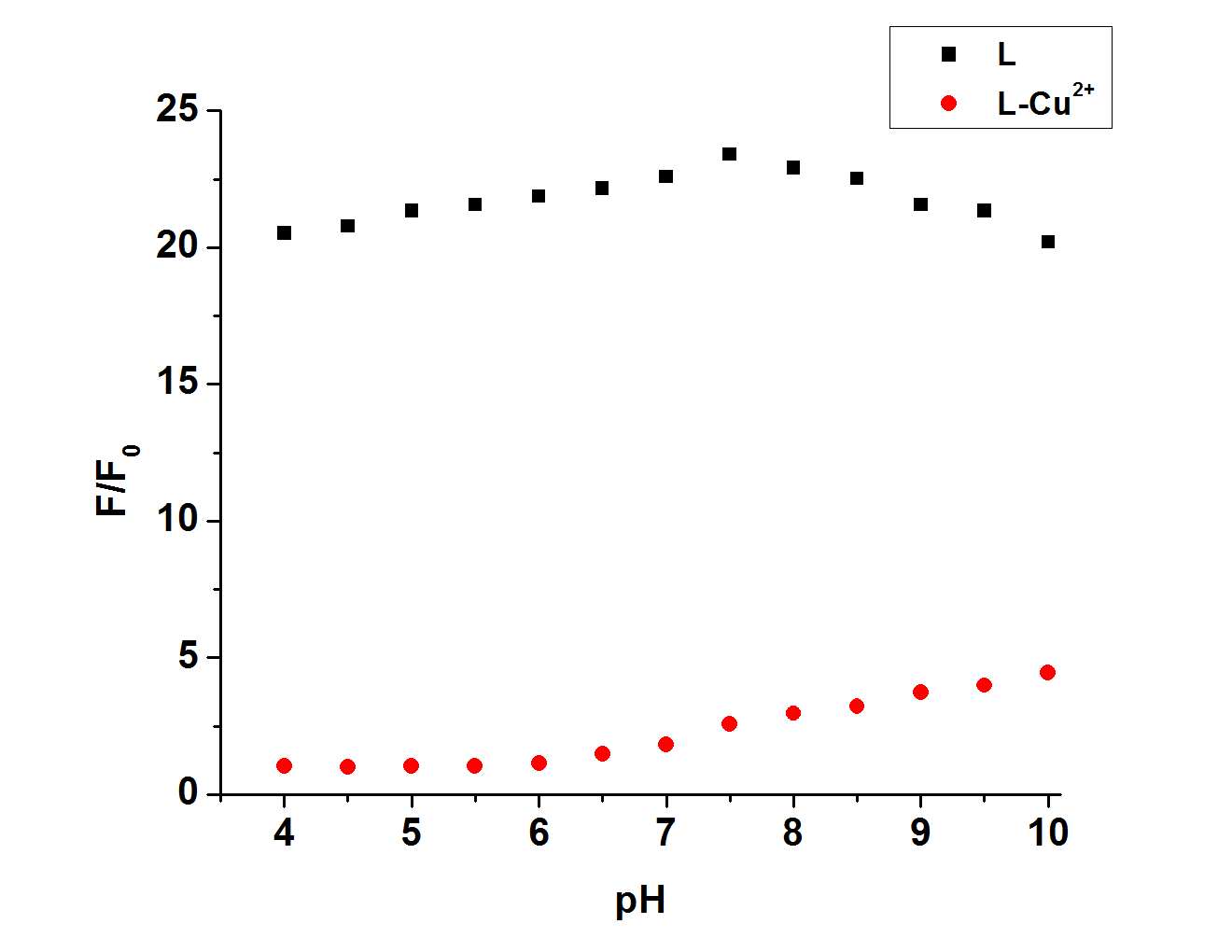

Supplement: S5 Fig — Fluorescence intensity was recorded at 575 nm. (TIF) [file pone.0186994.s005.tif]

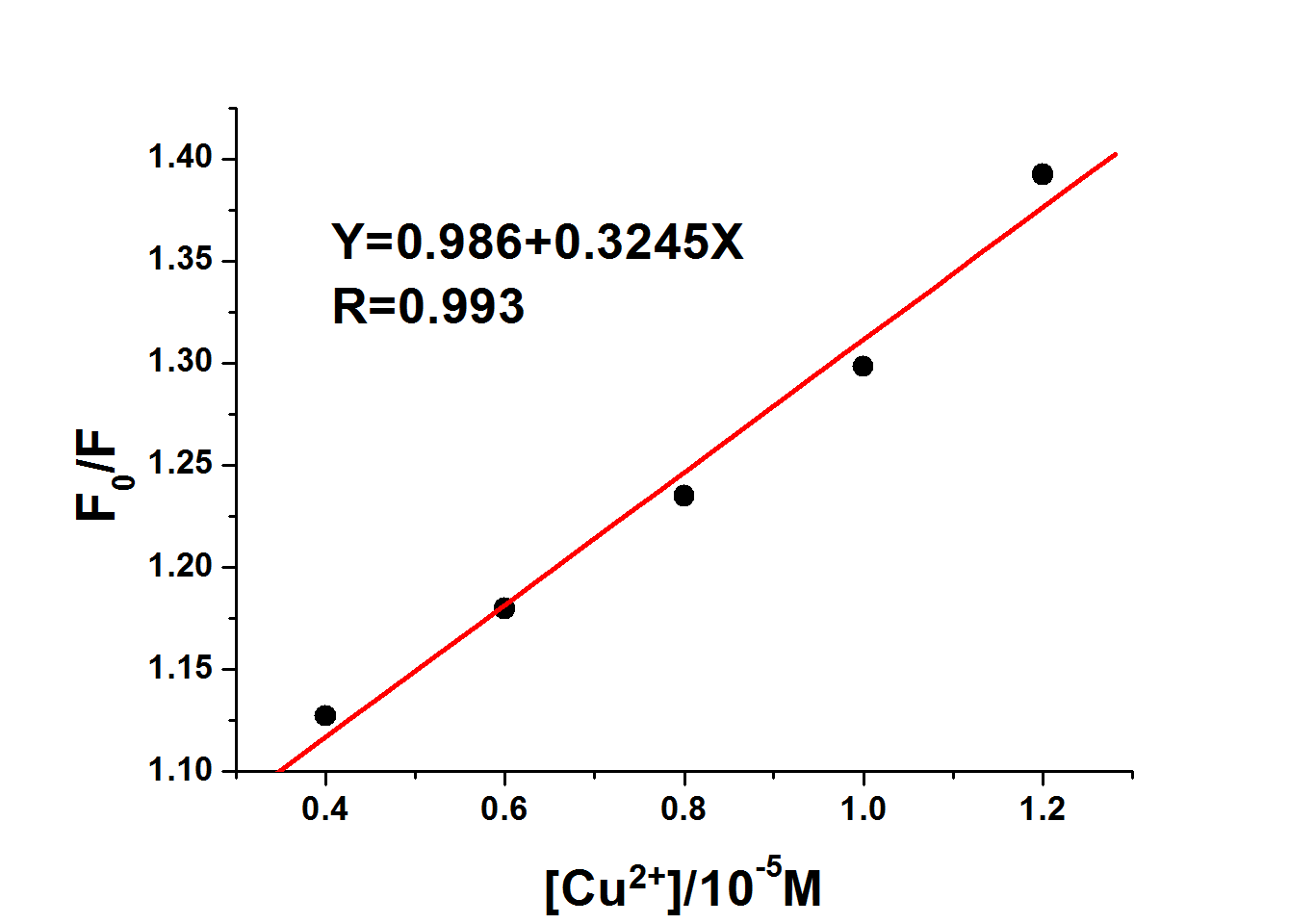

Supplement: S6 Fig — (TIF) [file pone.0186994.s006.tif]

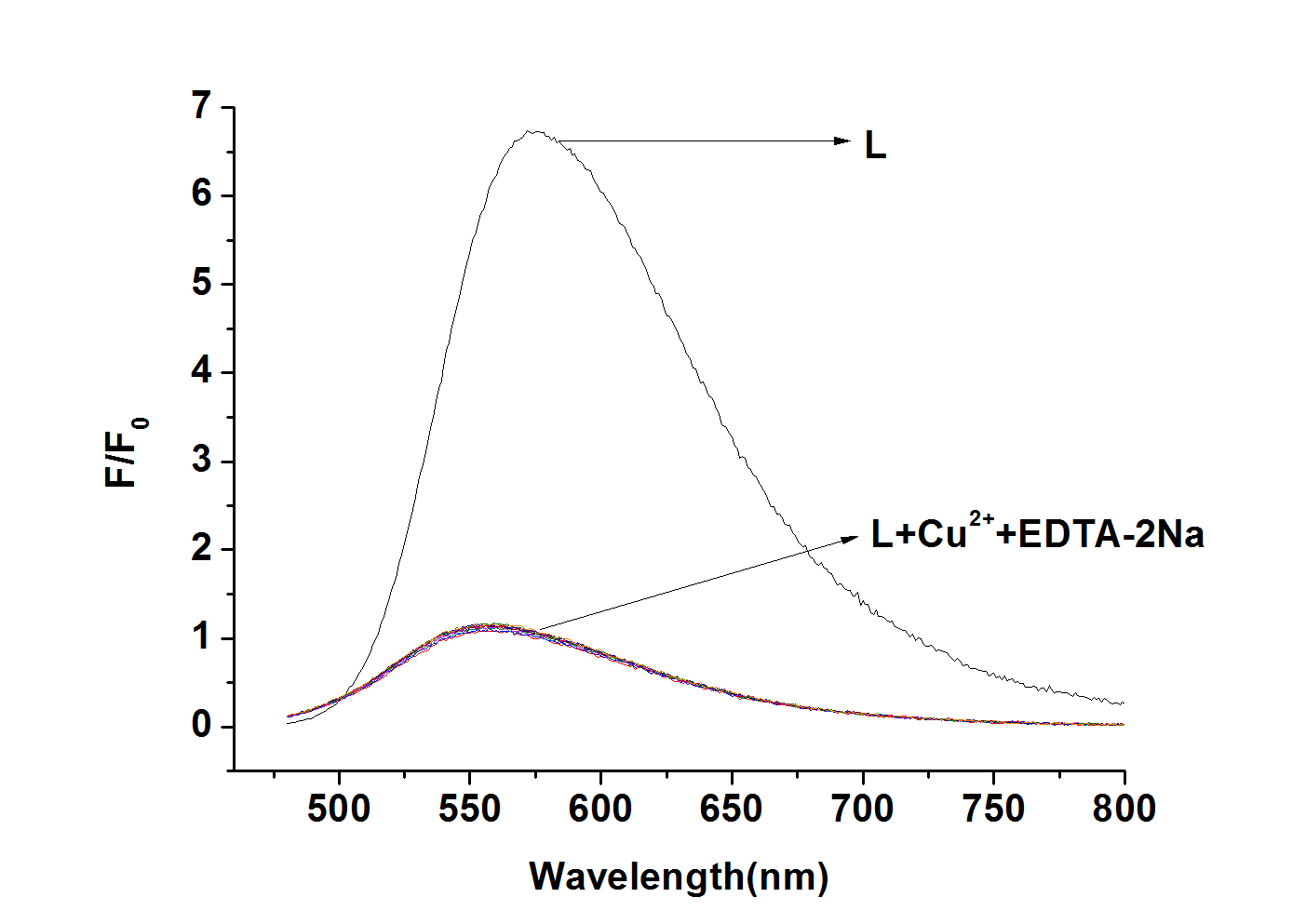

Supplement: S7 Fig — (TIF) [file pone.0186994.s007.tif]

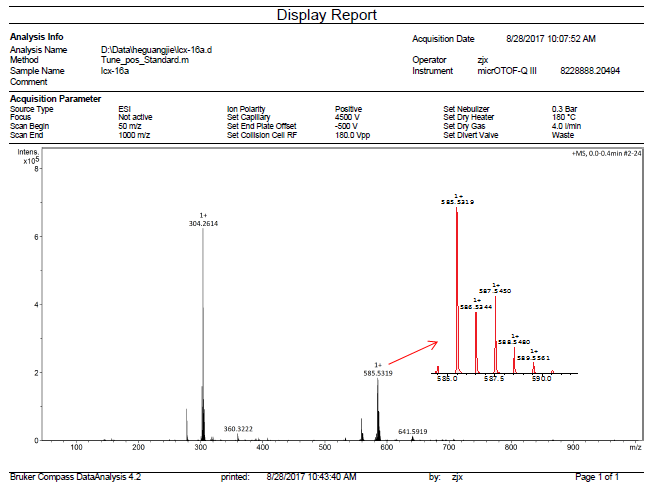

Supplement: S8 Fig — (TIF) [file pone.0186994.s008.tif]

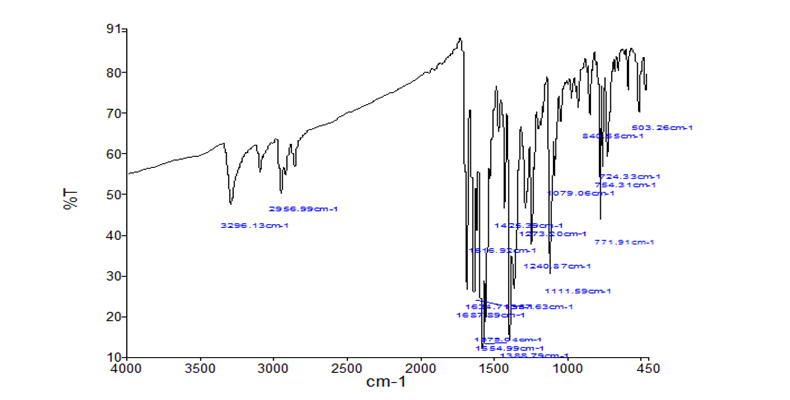

Supplement: S9 Fig — (TIF) [file pone.0186994.s009.tif]

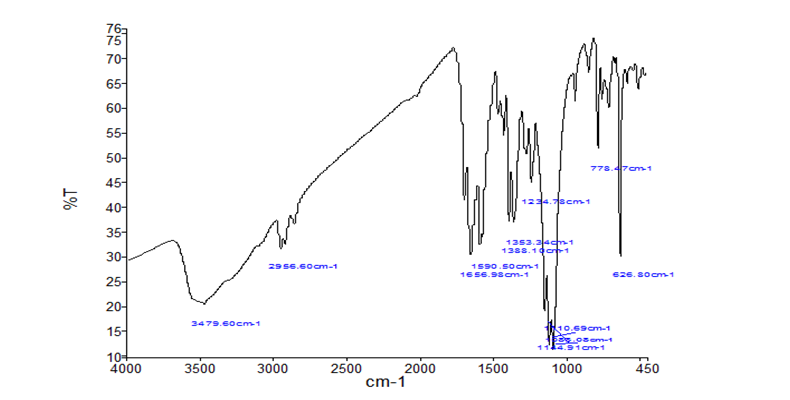

Supplement: S10 Fig — (TIF) [file pone.0186994.s010.tif]

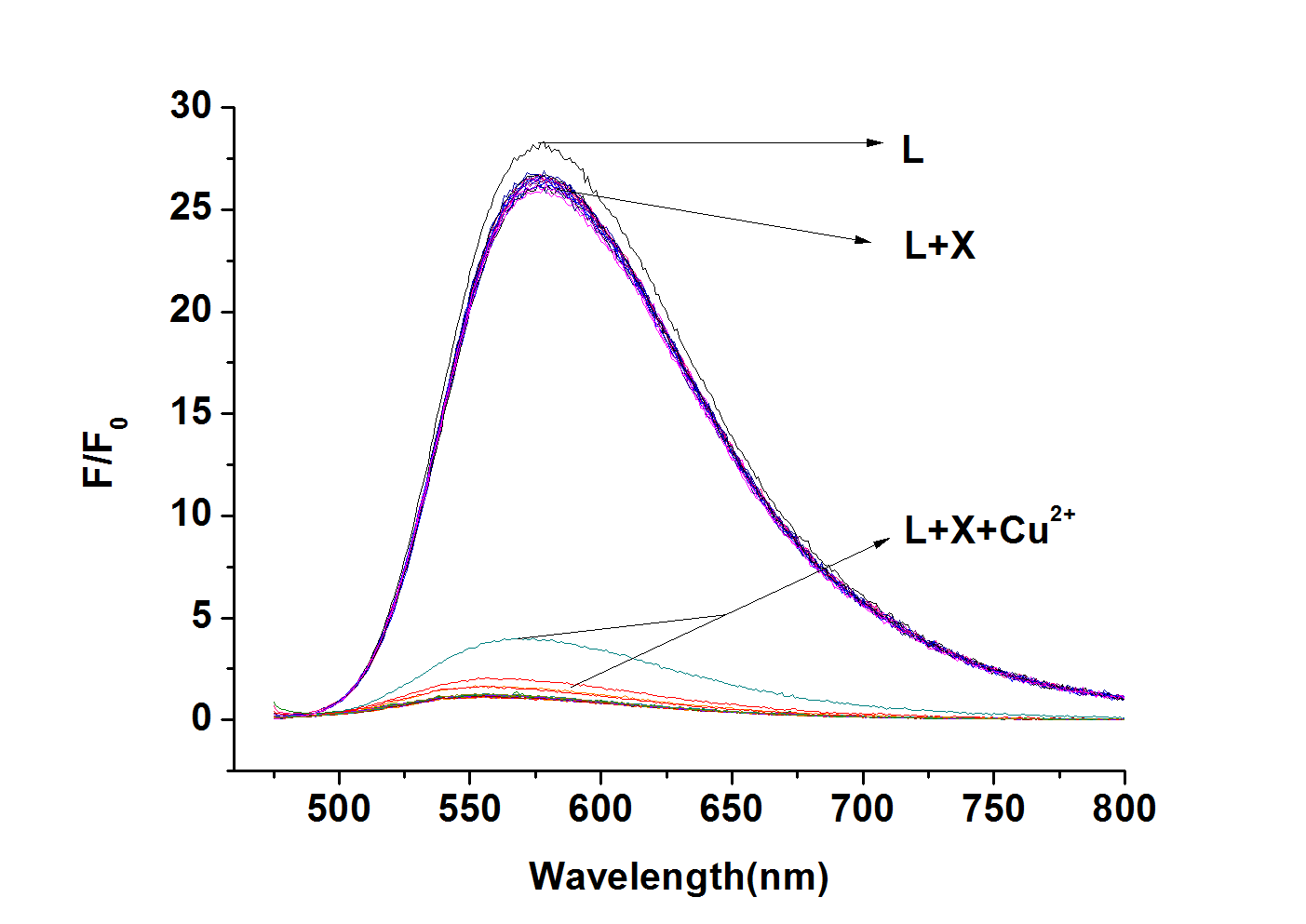

Supplement: S11 Fig — (TIF) [file pone.0186994.s011.tif]

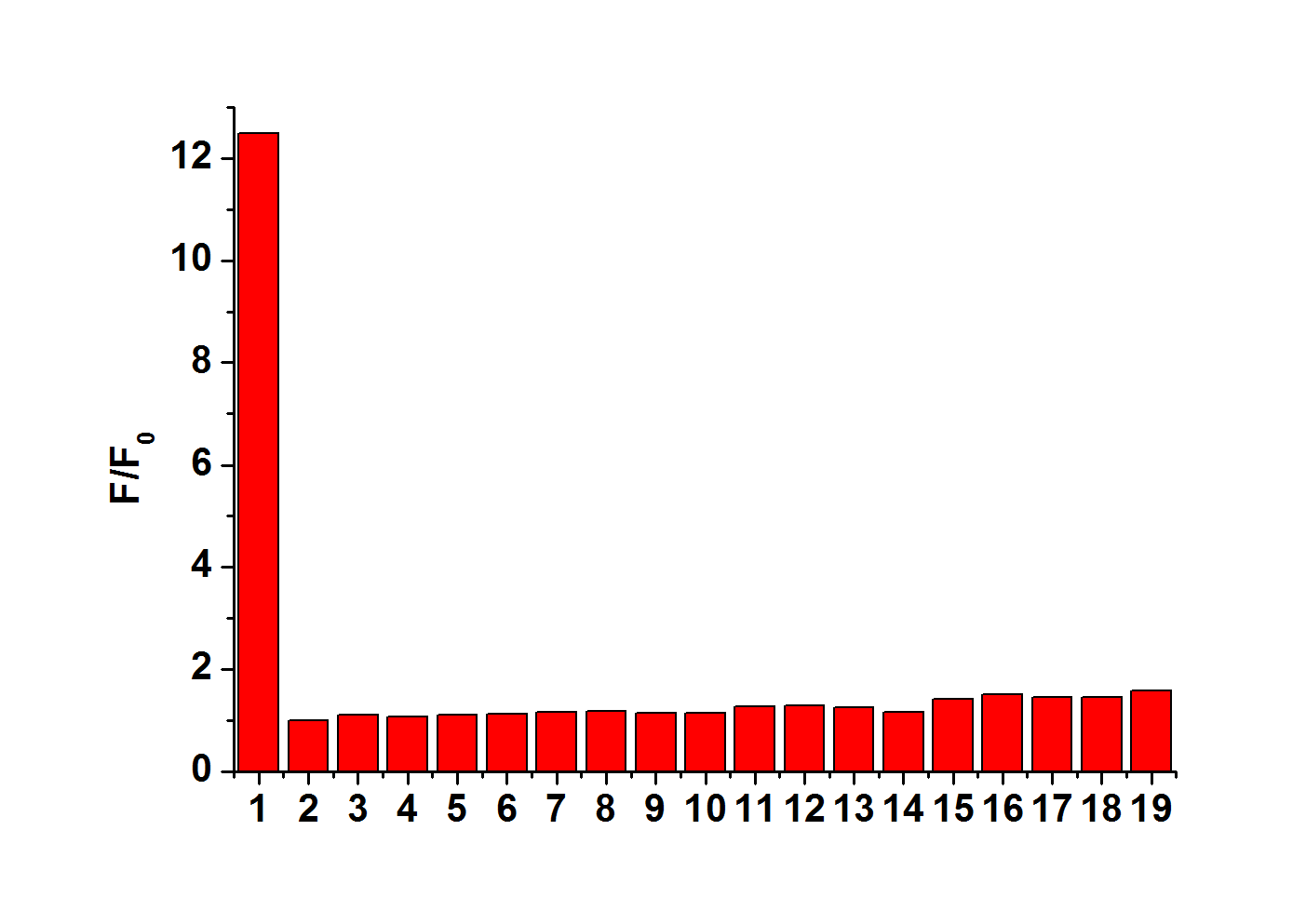

Supplement: S12 Fig — (1)L, (2)L-Cu2+, (3)F-, (4)Cl-, (5)Br-, (6)I-, (7)NO3-, (8)SO42-, (9)SO32-, (10)HSO3-, (11)PO43-, (12)HPO42-, (13)H2PO4-, (14)CO32-, (15)HCO3-, (16)CH3COO-, (17)PPi, (18)SCN-, (19)S2-. (TIF) [file pone.0186994.s012.tif]
